# Near-infrared fluorescent northern blot

**DOI:** 10.1261/rna.068213.118

**Published:** 2018-12

**Authors:** Bret R. Miller, Tianqi Wei, Christopher J. Fields, Peike Sheng, Mingyi Xie

**Affiliations:** 1 Department of Biochemistry and Molecular Biology, University of Florida, Gainesville, Florida 32610, USA; 2 UF Health Cancer Center, University of Florida, Gainesville, Florida 32610, USA; 3 UF Genetics Institute, University of Florida, Gainesville, Florida 32610, USA

**Keywords:** northern blot, near-infrared fluorescence, IR dye, RNA detection, nonradioactive

## Abstract

Northern blot analysis detects RNA molecules immobilized on nylon membranes through hybridization with radioactive ^32^P-labeled DNA or RNA oligonucleotide probes. Alternatively, nonradioactive northern blot relies on chemiluminescent reactions triggered by horseradish peroxidase (HRP) conjugated probes. The use of regulated radioactive material and the complexity of chemiluminescent reactions and detection have hampered the adoption of northern blot techniques by the wider biomedical research community. Here, we describe a sensitive and straightforward nonradioactive northern blot method, which utilizes near-infrared (IR) fluorescent dye-labeled probes (irNorthern). We found that irNorthern has a detection limit of ∼0.05 femtomoles (fmol), which is slightly less sensitive than ^32^P-Northern. However, we found that the IR dye-labeled probe maintains the sensitivity after multiple usages as well as long-term storage. We also present alternative irNorthern methods using a biotinylated DNA probe, a DNA probe labeled by terminal transferase, or an RNA probe labeled during in vitro transcription. Furthermore, utilization of different IR dyes allows multiplex detection of different RNA species. Therefore, irNorthern represents a more convenient and versatile tool for RNA detection compared to traditional northern blot analysis.

## INTRODUCTION

Northern blot analysis allows for identification and quantification of RNA molecules. Unlike methods such as reverse transcription PCR (RT-PCR) and RNase protection assay, northern blot analysis has the ability to distinguish RNA molecules based on size (Streit et al. 2009). This feature makes northern blot one of the most prominent methods for analyzing gene expression. The general workflow of a northern blot is: (i) RNA samples are separated by electrophoresis on a denaturing gel based on their sizes. (ii) RNAs are then transferred to a nylon membrane where they are subsequently cross-linked by 254 nm UV light. (iii) The membrane undergoes hybridization with a ^32^P-labeled oligonucleotide probe that is complementary to the RNA of interest. (iv) After hybridization, the membrane is exposed to an X-ray film or phosphor screen for detection of the radioactive signal.

The standard northern blot protocol can be modified using differently labeled probes. Due to its strong sensitivity, the most conventional probe label is radioactive ^32^P (Rio 2014). While the ^32^P-Northern technique is very sensitive, its use is often restricted due to the various regulations and safety precautions required (Jones 2005). Furthermore, its short half-life (∼14 d) limits reuse of ^32^P-labeled probes (Unterweger 2002). To address these issues, multiple nonradioactive northern blot methods have been developed (Kessler 1994). The most commonly used nonradioactive probes are Digoxigenin (DIG)-labeled or biotin-labeled (Holtke et al. 1995; Huang et al. 2014). However, detection of these probes relies on secondary recognition by an antibody or streptavidin conjugated with horseradish peroxidase (HRP), followed by a chemiluminescent reaction as indirect readout.

Recently, the use of near-infrared (IR) dyes, which have emission wavelengths in the spectrum of 650–900 nm, has become increasingly important in the detection of biomolecules (Guo et al. 2014). Low autofluorescence background and high signal-to-noise ratio in the near-infrared spectrum has prompted various new techniques using IR dyes for biomolecule detection both in vitro and in vivo (Owens et al. 2016). Specifically, IR dye-conjugated secondary antibodies have been widely used in western blot analysis (Weldon et al. 2008). In addition, several studies have used IR dye-labeled streptavidin or α-DIG antibody in Southern blot and northern blot analyses where probes are biotinylated or DIG-labeled (Zavala et al. 2014; MacDiarmid et al. 2016). Nonetheless, in these protocols, the IR dye is not covalently attached to the probe and the sensitivity has not been directly compared with ^32^P-Northern.

Here, we describe a novel nonradioactive method for northern blot analysis using IR dye-labeled probes. By incorporation of an azide (-N_3_)-modified nucleotide in the DNA probe, copper-free click chemistry can be used to covalently link the azide with the Dibenzocyclooctyl (DBCO) IR dye. This allows for highly efficient and stable incorporation of the IR dye into the probe (Zarnegar et al. 2016). The IR dye-labeled probes can be used in place of ^32^P-labeled probes for direct northern blot analysis, which we refer to as irNorthern. Compared to the previously described method of using the combination of biotinylated DNA probes and streptavidin-IR dyes (MacDiarmid et al. 2016), irNorthern using IR dye-labeled DNA probes is more sensitive and easier to perform. Furthermore, an irNorthern DNA probe could be prepared by terminal transferase (TdT) in the presence of azide-modified dUTP and an RNA probe could be generated by T7 in vitro transcription in the presence of azide-modified UTP, followed by DBCO IR dye labeling. Additionally, the use of a variety of IR dyes that emit at different wavelengths allows the simultaneous analysis of multiple RNA species.

## RESULTS

### irNorthern blot using DNA probe labeled with IR dye

**FIGURE 1.**
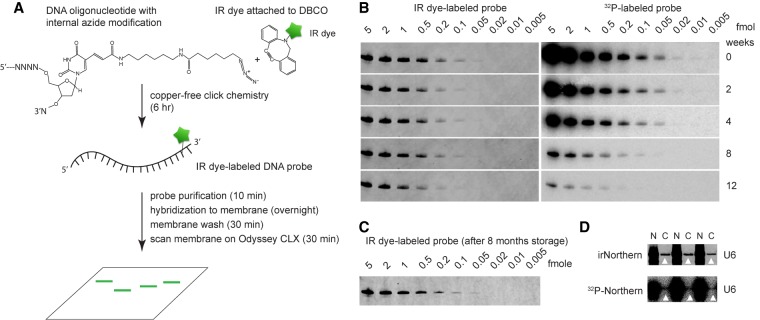
(*A*) Schematic diagram of IR dye labeling of azide-modified ssDNA probe followed by northern blot analysis. Time used on each step is indicated in the parentheses. (*B*) Northern blot analyses of serial diluted *Trichoplax adhaerens* (Ta) U6 snRNA samples for comparison of IR dye-labeled probe with ^32^P-labeled probe. In the *left* panel, the membrane was hybridized with IR dye-labeled probe and scanned on an Odyssey CLX for IR fluorescence detection. In the *right* panel, the same membrane was hybridized with ^32^P-labeled probe, exposed to a phosphor screen, and scanned on a Storm phosphorimager. The membrane was stripped after each scan and was rehybridized with the same diluted probes after 2, 4, 8, and 12 wk. (*C*) irNorthern analysis of Ta U6 using an IR dye-labeled ssDNA probe stored at −80°C for 8 mo prior to its usage. (*D*) Northern blot analyses of human U6 snRNA in nuclear (N) and cytoplasmic (C) RNAs extracted from HEK 293T cells, using an IR dye-labeled probe or a ^32^P-labeled probe. White triangles point to the U6 RNAs detected in the cytoplasmic fractions.

To develop irNorthern, we labeled a single stranded (ss)DNA probe with an IR dye using a protocol modified from Zarnegar et al. (2016) (Fig. 1A). A 30 nucleotide (nt) antisense ssDNA probe with an azide-modified thymidine was synthesized complementary to U6 small nuclear (sn)RNA. Subsequently, copper-free click chemistry was used to attach the IRDye 800CW DBCO to the probe. The labeled ssDNA probe was then purified and diluted to 1 nM in hybridization solution for the northern blot analysis.

The sensitivity of IR dye-labeled DNA probes in a northern blot was measured by probing a series of in vitro transcribed *Trichoplax adhaerens* (Ta) U6 snRNAs. Based on the analyses of five independent experiments, IR dye-labeled probes are capable of detecting RNA as low as 0.02–0.05 fmol (Fig. 1B; Supplemental Fig. S1). This sensitivity is comparable to that of the nonradioactive “locked nucleic acids (LNA), 1-ethyl-3-(3-dimethylaminopropyl), and digoxigenin” (LED) method which has been shown to detect 0.05 fmol of small RNAs (Kim et al. 2010). For comparison, we examined the same Ta U6 snRNA blots with ^32^P-labeled probes. We found that the ^32^P-labeled probe has a lower detection limit at 0.005–0.01 fmol (Fig. 1B; Supplemental Fig. S1). Therefore, irNorthern is slightly less sensitive compared to ^32^P-Northern.

We next compared the longevity of the IR dye-labeled probes to ^32^P-labeled probes, whose half-life is ∼14 d (Unterweger 2002). The Ta U6 snRNA northern membrane was reprobed after 2, 4, 8, and 12 wk using the same diluted IR dye-labeled probe or ^32^P-labeled probe. The sensitivity of the ^32^P-labeled probe diminished rapidly as expected (Fig. 1B, right panel). In contrast, the IR dye-labeled probe remained stable over the course of the experiment (Fig. 1B, left panel), and suggested that it is suitable for repeated use and long-term storage. We further confirmed that the IR dye-labeled probe maintains its sensitivity after 8 mo storage at −80°C (Fig. 1C).

By comparing the ^32^P-Northern and irNorthern blots, we also noticed that strong signals from the ^32^P-Northern tend to obscure weak signals in the neighboring lanes, while the strong IR signal is relatively contained (Fig. 1B; Supplemental Fig. S1). To illustrate this advantage for irNorthern, we probed for U6 snRNA on a northern blot with intervening lanes of nuclear and cytoplasmic RNAs from human embryonic kidney cells (Fig. 1D). Despite the strong nuclear U6 signal in neighboring lanes, irNorthern clearly detects trace amounts of possibly contaminating U6 in the cytoplasmic fractions. In contrast, the weak cytoplasmic U6 signal is completely overshadowed by the nuclear U6 signal in ^32^P-Northern. Therefore, irNorthern is suitable for detecting small amount of RNAs despite overwhelming nearby signals.

### irNorthern blot using alternative DNA and RNA probes

**FIGURE 2.**
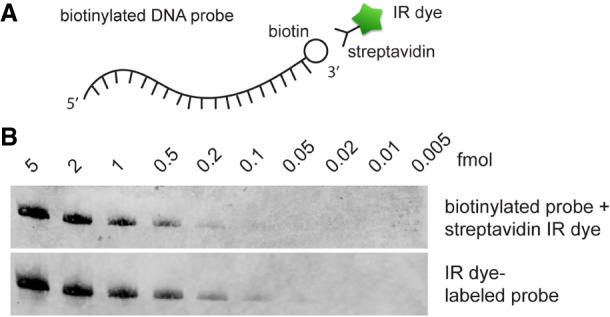
(*A*) Schematic diagram for a 3′ biotinylated ssDNA probe interacting with a streptavidin-IR dye. (*B*) Serial diluted Ta U6 snRNA samples were analyzed by irNorthern using a biotinylated probe conjugated with a streptavidin-IR dye or an IR dye-labeled probe.

Multiple northern blot methods utilize biotinylated probes and streptavidin conjugated with HRP or IR dye, due to the strong biotin-streptavidin interaction (Huang et al. 2014; MacDiarmid et al. 2016). However, streptavidin-IR dyes are indirectly conjugated on the northern probe in this case, and RNA detection requires a second incubation step (see Materials and Methods). We sought to compare the sensitivity of biotinylated probe conjugated to a streptavidin-IR dye and the IR dye-labeled probes in irNorthern (Fig. 2A). Utilizing the serial dilution of in vitro transcribed Ta U6 snRNA samples as in Figure 1, a biotinylated DNA probe was hybridized to the membrane overnight followed by incubation with IRDye 800CW streptavidin. Our results indicate that irNorthern with a biotinylated probe conjugated to streptavidin-IR dye provides about 1/4 the signal strength compared to an IR dye-labeled probe (Fig. 2B). However, using a biotinylated DNA probe for irNorthern presents a useful alternative, considering that biotinylated DNA probes could be prepared in-house by asymmetric PCR or primer extension in the presence of biotinylated dNTPs.

**FIGURE 3.**
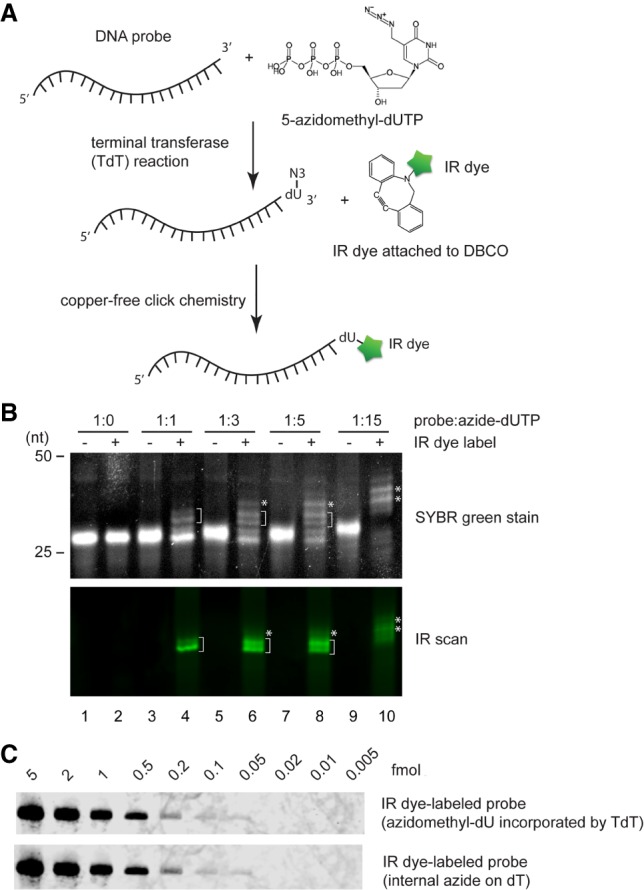
(*A*) Schematic diagram for preparing an IR dye-labeled DNA probe via terminal transferase (TdT) reaction. (*B*) Visualization of 5-azidomethyl-dU-incorporated and IR dye-labeled DNA probes by SYBR green staining and IR scan. In the TdT reactions, different DNA probe:5-azidomethyl-dUTP ratios were used as indicated on *top*. DNA probes containing one or two IR dyes were bracketed. The asterisks indicate the self-quenching probes. (*C*) Serial diluted Ta U6 snRNA samples were analyzed by irNorthern using probes containing TdT transferred 5-azidomethyl-dU or chemically synthesized probes containing azide-modified dT.

The probes used in prior irNorthern experiments were labeled with one IR dye at the azide-modified dT nucleotide. We asked if a probe labeled with multi-IR dyes could result in higher sensitivity. Because it is currently costly and therefore impractical to include many azide-modified dT residues in a single probe by chemical synthesis, we developed a protocol to attach multiple 5-azidomethyl-dU into a DNA probe using terminal transferase (TdT) (Fig. 3A). By adjusting the molar ratio between DNA probe and 5-azidomethyl-dUTP in the TdT reaction, we successfully attached multiple 5-azidomethyl-dU residues to the 3′ end of the probe, as visualized by SYBR green staining (Fig. 3B, SYBR green panel). However, when we labeled the probes with IRDye 800CW DBCO, only the probes including one or two 5-azidomethyl-dU were prominently detected (Fig. 3B, IR scan panel, bracketed bands). This was consistent with previous findings that coexistence of multiple IR dyes in close proximity results in self-quenching (Zhegalova et al. 2014). Self-quenching was most noticeable when the largest numbers of 5-azidomethyl-dUs were added, which led to the dimmest IR signal (Fig. 3B, lane 10). To minimize negative influence from unlabeled and self-quenched probes, we gel purified the probes labeled with only one or two IR dyes and performed irNorthern (Fig. 3B, lanes 6,8). The purified probes exhibit similar sensitivity compared to the IR dye-labeled probes containing chemically synthesized internal azide-dT (Fig. 3C), confirming the efficacy of our TdT-mediated IR probe preparation protocol.

Another commonly used probe in northern blot is the antisense RNA probe (Srivastava and Schonfeld 1991). RNA probes are generally transcribed in vitro in the presence of [α-^32^P] NTP and can have higher specificity due to their long lengths (Melton et al. 1984). Similarly, an IR dye-labeled UTP, Aminoallyl-UTP-ATTO-680, has been used to transcribe IR-fluorescent RNA in vitro (Köhn et al. 2010). However, transcription involving Aminoallyl-UTP-ATTO-680 is inefficient and the detection limit of the IR-fluorescent RNA was only about 1 fmol (Köhn et al. 2010). Accordingly, when we tried irNorthern using an ATTO-680-incorporated RNA probe antisense to Ta U6 snRNA, the detection limit was around 1 fmol (data not shown), much less sensitive compared to using an IR dye-labeled DNA probe (Fig. 1).

**FIGURE 4.**
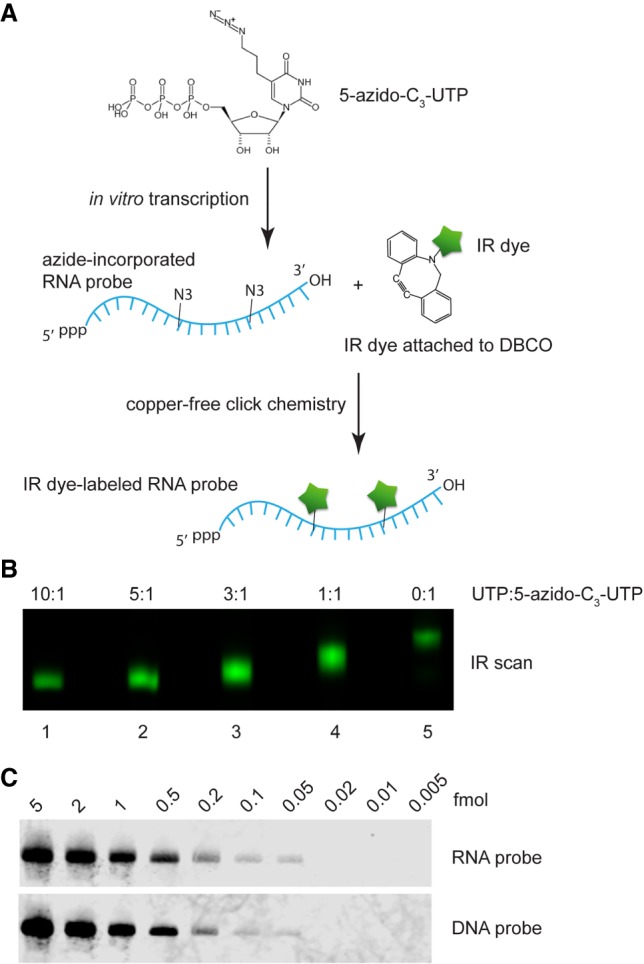
(*A*) Schematic diagram for preparing an IR dye-labeled RNA probe via T7 in vitro transcription. (*B*) Visualization of 5-azido-C_3_-U-incorporated and IR dye-labeled RNA probes by IR scan. In in vitro transcription reactions, different UTP: 5-azido-C_3_-UTP ratios were used as indicated on *top*. (*C*) Serial diluted Ta U6 snRNA samples were analyzed by irNorthern using either an IR dye-labeled RNA or DNA probe. Note that the irNorthern image for DNA probe is the same one used as the *bottom* panel in Figure 3C.

To develop an IR-fluorescent RNA probe with an alternative approach, we carried out in vitro transcription of antisense Ta U6 RNA using 5-Azido-C_3_-UTP, followed by IRDye 800CW DBCO labeling (Fig. 4A). Different UTP:5-Azido-C_3_-UTP ratios were tested. When all 30 Us in the RNA probe were azide-modified, IR dye labeling resulted in the largest mobility shift but the dimmest IR signal due to self-quenching (Fig. 4B, lane 5). We determined that using 3:1 UTP:5-Azido-C_3_-UTP resulted in an optimal Ta U6 RNA probe in terms of IR intensity (Fig. 4B). Ta U6 RNA was then analyzed by irNorthern using the RNA probe. As seen in Figure 4C, the RNA probe has a similar detection limit at 0.05 fmol as for the DNA probe. Note that the lower background for the RNA probe relative to the DNA probe may result from the higher hybridization temperature and more stringent washing conditions (see Materials and Methods). Therefore, we have developed a sensitive irNorthern protocol utilizing an IR dye-labeled RNA probe.

### Multiplexing in irNorthern blot analysis

**FIGURE 5.**
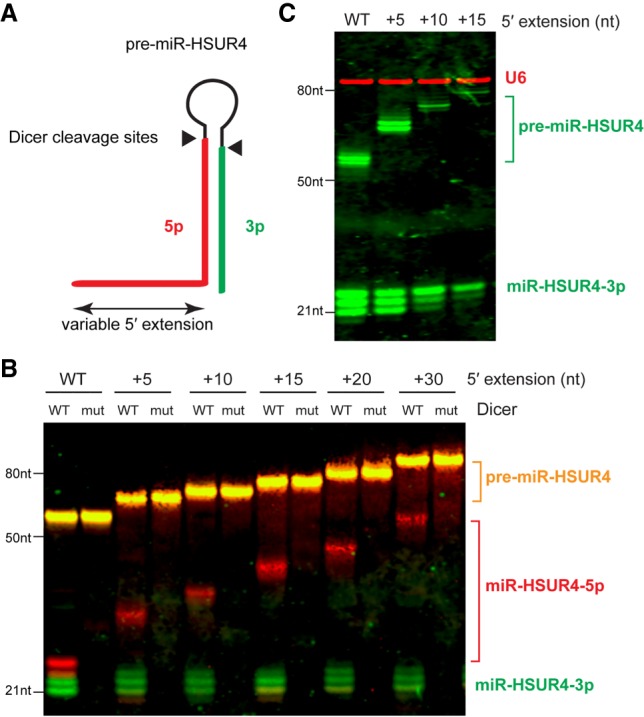
(*A*) Schematic diagram of Dicer cleavage on 5′-extended pre-miR-HSUR4. Dicer cleavage sites are indicated with black triangles. 5p and 3p miR-HSUR4s released from Dicer cleavage are highlighted in red and green, respectively. (*B*) Equal numbers of in vitro transcribed 5′-extended pre-miR-HSUR4s were processed with wild-type (WT) or mutant (mut) human Dicer and analyzed by multiplex irNorthern blot to detect miR-HSUR4-5p or -3p. The 5p miRNA probe was labeled with IRDye 800CW, and the detected miR-HSUR4-5p bands are shown in red. The 3p miRNA probe was labeled with IRDye 680RD, and the detected miR-HSUR4-3p bands are shown in green. The pre-miRNAs, shown in orange, were detected by both the 5p and 3p probes. (*C*) Multiplex irNorthern analysis of total RNAs extracted from HCT116 colorectal cancer cells, which express pre-miR-HSUR4 with various 5′ extensions from transfected plasmids. U6 (red) was detected by an IRDye 800CW-labeled probe, while miR-HSUR4-3p and pre-miR-HSUR4 (green) were detected by an IRDye 680RD-labeled probe.

As there are a variety of IR dyes emitting at different wavelengths, it is possible to use IR dye-labeled probes for a multiplex northern blot, which could not be achieved by ^32^P- or chemiluminescent reaction-based northern techniques. To this end, we performed irNorthern on a series of 5′-extended precursor microRNA (pre-miRNA) hairpins processed by Dicer (Sheng et al. 2018). Dicer cleavage of pre-miR-HSUR4, a *Herpesvirus saimiri* pre-miRNA, releases mature miR-HSUR4s from both the 3p and the extended 5p arms of the hairpin (Fig. 5A). We performed multiplex irNorthern with mature 3p and 5p miR-HSUR4 probes labeled with IRDye 680RD and 800CW, respectively (Fig. 5B). As expected, various miR-HSUR4-3p, which were detected by the IRDye 680RD-labeled probe (green), remained 22 nt in length. Appearance of mature miR-HSUR4-3p was due to Dicer cleavage because a catalytic site mutant Dicer did not produce such product (Fig. 5B, mut Dicer lanes). On the other hand, IRDye 800CW-labeled probe (red)-detected miR-HSUR4-5p show increased length as the extension length of the pre-miRNA increases (compare +5, +10, +15, +20, +30 nt lanes to the WT lane), consistent with previous findings (Sheng et al. 2018). The pre-miRNAs can be hybridized with both the 3p and the 5p miRNA probes, and therefore exhibit orange color (Fig. 5B).

We further tested multiplex irNorthern with total RNAs extracted from HCT116 colorectal cancer cells, which express pre-miR-HSUR4 with various 5′ extensions from transfected plasmids (Sheng et al. 2018). Using an IRDye 680RD-labeled probe against miR-HSUR4-3p and an IRDye 800CW-labeled probe against U6 snRNA, both RNAs were detected on the same blot unambiguously (Fig. 5C). In conclusion, irNorthern is suitable for multiplexing on total RNA samples.

## DISCUSSION

The safety precautions for using radioactive probes in northern blot have prompted the design of nonradioactive detection techniques. Of note is the use of biotinylated or DIG-labeled probes in combination with HRP-mediated chemiluminescent reactions (Kim et al. 2010; Huang et al. 2014). Nonetheless, these northern blot techniques involve chemical reactions for secondary detection, making them relatively cumbersome to perform.

We developed a highly sensitive and straightforward nonradioactive northern blot technique utilizing azide-modified oligonucleotides labeled with DBCO IR dye (Fig. 1A). While labeling these probes requires less safety precautions compared to ^32^P labeling, they are still capable of detecting RNA samples as low as ∼0.05 fmol (Fig. 1B; Supplemental Fig. S1). Furthermore, IR dye-labeled probes can be stored and reused for extended periods of time (Fig. 1B). While we showed that an IR dye-labeled probe maintained its sensitivity after 8 mo storage in −80°C (Fig. 1C), it is expected that the probe can be stored even longer without compromising its sensitivity. As a result, researchers can save a considerable amount of time and resources, because highly sensitive IR dye-labeled probes do not have to be regenerated frequently, as in the case of using ^32^P-labeled probes. Additionally, IR dye-labeled probes can be used to detect multiple RNA species at the same time (multiplexing) (Fig. 5), which is not possible using ^32^P- or chemiluminescent reaction-based northern blot techniques.

While our multiplex irNorthern displayed its functionality in pre-miRNA processing, this method also has applications in other RNA processing reactions such as mRNA splicing (Hirose et al. 2006), in which the intron and the exon could be detected by different probes and the pre-mRNA could be detected by both probes. In addition, probe color could be adjusted by labeling with two different IR dyes to enable more versatile multiplexing. For instance, IRDye 680RD and 800CW could be mixed in a 1:1 ratio to label a probe as “orange” (Fig. 5B). With future advances in the accuracy of detecting subtle differences in IR fluorescence, the combination of multiple dyes at different ratios could distinguish dozens of RNAs simultaneously in one northern blot.

Previously described improvements on the northern blot method could be implemented with the use of IR dye-labeled probes to produce even lower detection limits. For example, replacing UV crosslinking with 1-ethyl-3-(3-dimethylaminopropyl) carbodiimide hydrochloride (EDC) crosslinking results in a 50-fold increase in small RNA detection (Pall and Hamilton 2008). Furthermore, incorporation of LNA into the probe strands also results in elevated detection of RNA (Válóczi et al. 2004). Similar to the LED method described by Kim et al. (2010), both EDC crosslinking and LNAs could be incorporated into our irNorthern protocol and may result in enhanced RNA detection.

We explored another possibility of increasing the irNorthern sensitivity by incorporating multiple dyes onto the probe. However, we estimated that coexistence of more than three dyes on a single probe severely reduced the IR intensity due to self-quenching (Figs. 3B, 4B). Such multi-IR dye-labeled probes failed to reach sensitivity that exceeds the ^32^P labeled probes, even though we were able to produce DNA and RNA probes labeled with hundreds of IR dyes (data not shown). However, improving IR technologies that minimize the self-quenching effect could allow for more sensitive irNorthern probes to be synthesized with ease in the future (Zhegalova et al. 2014).

## MATERIALS AND METHODS

### Preparation of IR dye-labeled DNA probes

IrNorthern DNA probes for U6: 5′-GCAGGGGCCATGCTAATCTTCTCTGTATCG/iAzideN/T-3′; miR-HSUR4-5p: 5′-TTATAGCTGTAGCAACACGGT/iAzideN/A-3′; and miR-HSUR4-3p: ACGTGTTGCCCACTGCTATAAA/iAzideN/A-3′ were synthesized by Integrated DNA Technology (IDT). To label the azide-modified oligonucleotides with IR dye, 2.5 nmol oligonucleotides were mixed with 50 nmol IRDye 680RD or 800CW DBCO (Li-Cor Biosciences) in phosphate buffer saline (137 mM NaCl, $10\;\text{mM}\;{\text{PO}}_{4}^{3-}$, 2.7 mM KCl, pH 7.4) at 25°C for 6 h. IR dye-labeled oligonucleotides were then purified by Microspin G-25 column (GE Healthcare) or Ampure XP beads (Beckman Coulter) according to the manufacturer's instructions. Minor modifications were introduced for Ampure XP beads purification of short oligonucleotides. Specifically, 2 volumes of beads and 5.4 volumes of isopropanol were mixed with the reaction. The mixture was incubated at room temperature for 15 min and placed on a magnet stand to separate the beads from the supernatant. The beads were washed twice with 85% ethanol, and the DNA was eluted with 20–50 µL of H_2_O.

The TdT reaction was performed in 30 µL containing 50 mM KOAc, 20 mM Tris-acetate pH 7.9, 10 mM MgOAc, 250 µM CoCl_2_, 20 U TdT (New England Biolabs, NEB), 100 pmol U6 DNA probe (5′-GCAGGGGCCATGCTAATCTTCTCTGTATCG-3′), and 100 to 1500 pmol of 5-azidomethyl-dUTP (Jena Bioscience). After incubation at 37°C for 1 h, the reaction was purified by Ampure XP beads, labeled with IRDye 800CW DBCO, and purified again by Ampure XP beads as described above. Labeled probes were separated on an 8 M Urea 15% acrylamide gel. After IR scan and SYBR green imaging (Fig. 3B), the desired probes were purified from the gel.

### Preparation of RNA probes for irNorthern

To synthesize antisense RNA probes for *Trichoplax* U6, PCR templates containing a T7 promoter were used in T7 run-off transcription reactions. Each 10 µL reaction containing 40 mM Tris-HCl pH 8.0, 25 mM NaCl, 2 mM Spermidine(HCl)_3_, 8 mM MgCl_2_, 1 mM ATP, 1 mM UTP, 1 mM GTP, 1 mM CTP, 2 U/µL Murine RNase Inhibitor (NEB), 10 mM DTT, and 0.4 U/µL T7 RNA polymerase was incubated at 37°C overnight. In the transcription reactions including Aminoallyl-UTP-ATTO-680, 1 mM UTP was replaced by 50 µM UTP and 50 µM Aminoallyl-UTP-ATTO-680 (Jena Bioscience). In the reactions including 5-Azido-C_3_-UTP, 1 mM UTP was replaced by 1 mM total UTP with different ratios of UTP:5-Azido-C_3_-UTP (Jena Bioscience) as indicated in Figure 4B. In vitro transcribed RNAs were purified by 8 M Urea 6% acrylamide gel and resuspended in H_2_O. The 5-Azido-C_3_-U containing RNA probes were labeled with IRDye 800CW DBCO and purified by Ampure XP beads using the conditions described above.

### Preparation of 5′ end ^32^P-labeled probes

Ten picomoles of U6 DNA probe was incubated with 150 µCi 6000 Ci/mmol γ-^32^P-ATP (PerkinElmer), 10 U T4 PNK (NEB) in 70 mM Tris-HCl, 10 mM MgCl_2_, 5 mM DTT, pH 7.6 at 37°C for 30 min. The ^32^P-labeled oligonucleotides were then purified by a Microspin G-25 column according to the manufacturer's instructions.

### Northern blot analysis

In vitro transcribed Ta U6 RNAs were electrophoresed in an 8 M Urea 10% acrylamide gel and then transferred to a Hybond N+ membrane (Amersham). The membrane was cross-linked twice with 254 nm UV light at 120 mJ/cm^2^ using a Stratalinker UV crosslinker 2400 (Agilent genomics). After prehybridization with 10 mL ExpressHyb Hybridization Solution (Clonetech) for 30 min at 37°C, the membrane was hybridized with 10 pmol IR dye-labeled oligonucleotide U6 probe in 10 mL ExpressHyb solution at 37°C overnight. The membrane was washed with 2× SSC, 0.1% SDS (10 min at room temperature), 1× SSC, 0.1% SDS (10 min at room temperature) and analyzed by a Li-Cor Odyssey CLX scanner. The membranes used for irNorthern in Figure 1D and Figure 5 were prepared previously (Sheng et al. 2018). For miR-HSUR4-5p and -3p multiplex probing, the hybridization temperature was set at 45°C to prevent annealing between the two highly complementary probes and favor annealing between the probes and the RNA targets.

For northern blots using a ^32^P-labeled DNA probe, the protocol was the same as irNorthern, except that the washed membrane was exposed to a phosphor screen overnight and scanned on a Storm phosphorimager (Amersham). In between probing with different DNA probes, the membranes were stripped with microwave-boiled 0.1× SSC, 1% SDS, 40 mM Tris-HCl pH 8.0 for 10 min twice. The membranes were rescanned to ensure the probes were successfully removed. When comparing the sensitivity of IR dye-labeled probes to ^32^P labeled probes, we alternated the first probe applied to the membrane in five different experiments and found that probing the membrane first with either the IR dye-labeled or the ^32^P-labeled probe did not affect our conclusions (Fig. 1B; Supplemental Fig. S1).

For irNorthern using biotinylated DNA probes, after overnight hybridization and brief washes of 2× SSC, 0.1% SDS (2 min at room temperature), and 1× SSC, 0.1% SDS (2 min at room temperature) the membranes were incubated with 1:10,000 diluted IRDye 800CW streptavidin in ExpressHyb solution at 30°C for 30 min and then washed and analyzed as described above.

For irNorthern using an RNA probe, the hybridization was carried out at 65°C overnight. The membranes were washed with 2× SSC, 0.1% SDS (15 min at room temperature), 1× SSC, 0.1% SDS (15 min at room temperature) and 0.2× SSC, 0.1% SDS (15 min at room temperature) and analyzed as described above. Unlike the DNA probes, RNA probes cannot be completely stripped off the membrane.

As probes were continuously reused during experimentation, all IR dye-labeled probes diluted in hybridization solution were stored at −20°C while stock solutions of IR labeled probes were stored at −80°C. IR dyes and IR dye-labeled probes were protected from strong light exposure during irNorthern procedure and storage.

## SUPPLEMENTAL MATERIAL

Supplemental material is available for this article.

## Supplementary Material

Supplemental Material
