# Supplementary material for: Near-infrared fluorescent northern blot
Source: RNA. 2018 Dec;24(12):1871–7. doi: 10.1261/rna.068213.118 (PMC6239192; doi:10.1261/rna.068213.118)
Supplement: Supplemental Material [file supp_24_12_1871__index.html]

Near-infrared fluorescent northern blot — Supplemental Material 

# Near-infrared fluorescent northern blot

## Supplemental Material

- Supplemental\_Fig\_S1\_Legend.docx
- Supplemental\_Fig\_S1.tif
