# Supplementary material for: Near-infrared fluorescent northern blot
Source: RNA. 2018 Dec;24(12):1871–7. doi: 10.1261/rna.068213.118 (PMC6239192; doi:10.1261/rna.068213.118)
Supplement: Supplemental Material [file supp_068213.118_Supplemental_Fig_S1_Legend.docx]

**Supplemental Figure 1.** Replicate Northern blot analyses using IR dye-labeled probes vs ^32^P-labeled ssDNA probes. In addition to the membrane in Fig. 1B, four additional membranes with serial diluted Ta U6 snRNAs were prepared and hybridized with both IR dye-labeled probes and ^32^P-labeled probes. The graphs show quantitation of Ta U6 snRNA on Northern blots detected by an IR dye-labeled probe or a ^32^P-labeled probe. Relative intensities for each RNA sample (0.005 to 5 fmole) were derived from five Northern blot membranes in Fig. 1 and Fig. S1. Error bars represent standard deviations from the mean (n=5). A linear trendline was fitted with R^2^ value indicated.
