# Supplementary figures and images for: Near-infrared fluorescent northern blot
Source: RNA. 2018 Dec;24(12):1871–7. doi: 10.1261/rna.068213.118 (PMC6239192; doi:10.1261/rna.068213.118)

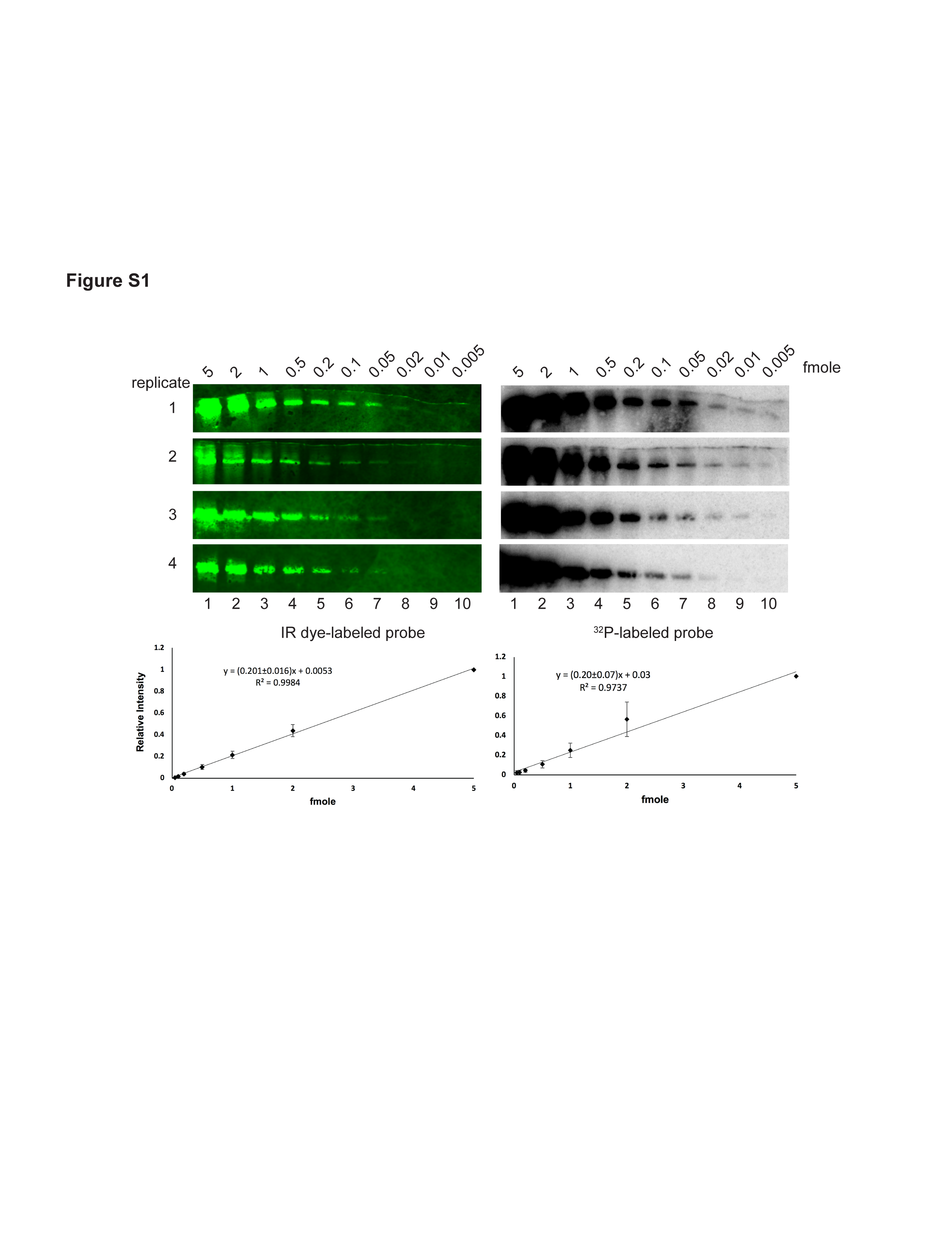

Supplement: Supplemental Material [file supp_068213.118_Supplemental_Fig_S1.tif]
